# Establishment of trimester-specific reference intervals of renal function tests and their predictive values in pregnant complications and perinatal outcomes: A population-based cohort study

**DOI:** 10.1016/j.plabm.2023.e00342

**Published:** 2023-10-16

**Authors:** Lican Han, Lin Liu, Lanlan Meng, Shaofei Su, Yifan Lu, Zhengwen Xu, Guodong Tang, Jing Wang, Hongyuan Zhu, Yue Zhang, Yanhong Zhai, Zheng Cao

**Affiliations:** a Department of Laboratory Medicine, Beijing Obstetrics and Gynecology Hospital, Capital Medical University, Beijing Maternal and Child Health Care Hospital, Beijing, China; b Center of Clinical Mass Spectrometry, Beijing Obstetrics and Gynecology Hospital, Capital Medical University, Beijing Maternal and Child Health Care Hospital, Beijing, China; c Central Laboratory, Beijing Obstetrics and Gynecology Hospital, Capital Medical University, Beijing Maternal and Child Health Care Hospital, Beijing, China; d Information Center, Beijing Obstetrics and Gynecology Hospital, Capital Medical University, Beijing Maternal and Child Health Care Hospital, Beijing, China; e Department of Clinical Laboratory, Beijing Chao-yang Hospital, Capital Medical University, Beijing, China

**Keywords:** Reference interval, Renal function test, Adverse pregnancy outcomes, Cohort study

## Abstract

**Objectives:**

In this study, we aimed to establish the trimester-specific RIs of renal function tests (RFTs) in singleton pregnant women and investigate the associations between adverse perinatal outcomes and abnormal renal function laboratory results.

**Methods:**

The results of RFTs and the associated medical records were retrieved from 16489 singleton pregnant women who underwent first- and third-trimester prenatal screening and gave a live birth at out institute between August 2018 and December 2019. The RFTs were performed on the automated immunochemistry platform ARCHITECT ci16200 (Abbott Laboratories Ltd, Abbott Park, Illinois, US) in the clinical laboratory of our institute. The nonparametric 2.5th-97.5th percentile intervals and the indirect Hoffmann methods were used to define the trimester-specific RIs. The associations between abnormal RFTs and adverse pregnancy outcomes was assessed statistically by logistic regression.

**Results:**

There was no significant difference between the direct observational and the indirect Hoffmann methods in establishing RIs of RFTs. Compared with RFTs in the first trimester, the concentrations of serum BUN and Crea were slightly decreased (*p* < 0.001), and the serum UA and Cys C levels were significantly elevated in the third trimester (*p* < 0.001). In the logistic regression analysis, high concentrations of UA, Crea, and Cys C in late pregnancy were associated with an increased risk of postpartum hemorrhage. Meanwhile, early pregnancy UA was associated with a modestly increased risk of GDM, GH, and PE.

**Conclusion:**

It is necessary to establish trimester-specific RIs for RFTs, in order to appropriately interpret laboratory results and to identify women with high risks of developing various adverse outcomes.

## Highlights

• There was no significant difference between the direct observational and the indirect Hoffmann methods in establishing RIs of RFTs.
• The serum concentrations of BUN and Crea were slightly decreased, and the UA and Cys C levels were significantly elevated in the third trimester.
• The third trimester UA, Crea, and Cys C were associated with an increased risk of PPH; the first trimester UA increased the risks of GDM, GH, and PE.

## 1 Introduction

Pregnancy is a unique and complex progression that leads to significant physiological changes in organ function [1,2], affecting almost every aspect of renal physiology [3]. For example, kidney and systemic hemodynamics show significant volume expansion and vasodilation. Compared to nonpregnant levels, glomerular filtration rate (GFR) increase by 50 %, and renal plasma flow (RPF) increase by 80 %, resulting in lower serum urea, uric acid, and creatinine levels [3,4]. Therefore, it is essential and recommended to establish reference intervals (RIs) for renal function tests (RFTs) at different gestational ages for proper interpretation of laboratory results during pregnancy [5]. The first objective of this study was to establish trimester-specific RIs for blood urea nitrogen (BUN), uric acid (UA), creatinine (Crea), and cystatin C (Cys C) with direct (by recruiting healthy subjects) and indirect (by statistical calculations) methods.

In addition, renal dysfunction during pregnancy may interfere with regular physiological changes, increasing the risk of adverse pregnancy outcomes [3,6]. For instance, Hellen et al. have shown that the serum level of cystatin C was higher in the group of pregnant women with pre-eclampsia than in normal pregnancy [7]. A risk relationship between abnormal renal function and higher bleeding tendencies was reported in some retrospective research [8,9]. In a study by Wolak et al., it was found that serum UA was an independent risk factor for GDM [10]. The second objective of this study is to statistically investigate the associations between RFTs and the risk of developing peri- and postpartum complications to meet clinical needs.

## 2 Methods

### 2.1 Patient selection and data extraction

In this observational cohort study, the RFTs laboratory results and relevant medical records of the singleton pregnant women who were registered for regular antenatal check-ups and gave a live birth at our institute were retrieved. In sum, from August 2018 to December 2019, a total of 16489 singleton pregnant eligible women with first- and third-trimester RFTs results and their complete perinatal medical records were included for the subsequent analysis. For the establishment of the trimester-specific RIs of BUN, UA, Crea and Cys C, the healthy pregnant women were selected using the following criteria: presenting normal pregnancy test results (i.e., routine blood, urine and biochemical tests) through the entire pregnancy and displaying no adverse or abnormal pregnancy complications and outcomes [11].

In our study, four pregnant complications including gestational hypertension (GH), gestational diabetes mellitus (GDM), preeclampsia (PE) and intrahepatic cholestasis of pregnancy (ICP), and two adverse perinatal outcomes including macrosomia and postpartum hemorrhage (PPH) were analyzed in the following research [12,13]. The definitions of these diseases or conditions were in Supplementary Table 1. This research protocol was approved by the Ethics Committee of Beijing Obstetrics and Gynecology Hospital (2022-KY-007-02).

### 2.2 Laboratory testing

All the RFTs assays were conducted with 2 ml serum collected from subjects after 8–10 h of fasting on the automated immunochemistry platform ARCHITECT ci16200 (Abbott Laboratories Ltd, Abbott Park, Illinois, US) in the clinical laboratory of our institute. The following reagents that were compatible with the analyzer were used in renal function testing: Urea assay kit (60120UN22, Abbott Park, IL, 60064USA), UA assay kit (80591UN21, Abbott Park, IL, 60064USA), Cr Enz assay kit (20268Y600, Abbott Park, IL, 60064USA) and Cys C assay kit (22–0424, Strong Biotechnologies, China).

The limits of detection for serum BUN, UA, Crea, and Cys C were 0.25 mmol/L, 5.3 μmol/L, 8.8 μmol/L, and 0.05 mg/L, respectively. The intra-assay coefficients of variation (CV) of serum BUN, UA, Crea, and Cys C were 3.4 %, 2.9 %, 3.1 %, and 5.2 %, respectively. The inter-assay CV were 6.8 %, 5.7 %, 6.2 %, and 10.4 %, respectively.

### 2.3 Statistical analysis

The data were assessed for normality using the Kolmogorov-Smirnov method, which showed all non-normally distributed. Non-normally distributed variables were expressed as medians and percentiles, and the Mann‒Whitney *U* test was used to compare the differences between groups for statistical significance in the levels of RFTs between the early and late stages of pregnancy. The 2.5th and 97.5th percentiles (the nonparametric approach) were calculated as the RIs for BUN, UA, Crea, and Cys C that were derived from healthy pregnant women. Meanwhile, the indirect Hoffmann [14] method was applied to calculate the RIs of RFTs in singleton pregnancy (n = 16489). The detailed steps for the estimation of RIs with the Hoffmann method can be found in the protocol published previously [14,15]. To determine the statistical significance of the differences between directly measured RI and the indirectly estimated RI, the reference change value (RCV) was calculated. Their difference is significant only if it is greater than RCV. All data analysis was performed using the IBM SPSS Statistic 25 (SPSS, Inc., Chicago, IL, USA).

In this study, logistic regression was performed to estimate the odds ratio (OR) and 95 % confidence interval (CI) for the adverse pregnancy outcomes, and maternal age and pre-pregnancy body mass index (BMI) were adjusted for confounding factors to analyze the relevance of the correlation between abnormal renal function and adverse pregnancy outcomes [16].

## 3 Result

### 3.1 Trimester-specific RIs for renal function tests (RFTs)

**Fig. 1:**
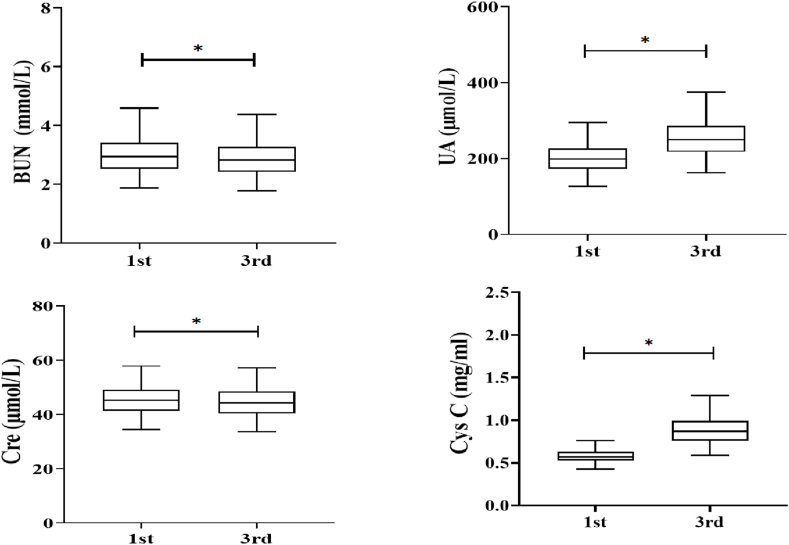
The box plots representing RFTs levels in healthy pregnant women in first and third trimesters of singleton pregnancies. *Indicates: *p* < 0.001; BUN, blood urea nitrogen; UA, uric acid; Crea, creatinine; Cys C, cystatin C.

According to the CLSI guideline C28-A3 [17], the laboratory results of both the first- and third-trimester RFTs from the same subjects were included from 4206 healthy pregnant women meeting the selection criteria for the RIs establishment. The overall data distribution for BUN, UA, Crea, and Cys C were presented with box plots in Fig. 1. Slightly decreased serum concentrations of BUN and Crea were observed in the third-trimester than those in the first trimester (*p* < 0.001). By contrast, the serum UA and Cys C levels were significantly increased in the third-trimester when compared with those in the first trimester (*p* < 0.001). The trimester-specific RIs for RFTs determined with nonparametric analysis (n = 4206) were shown in Table 1. As expected, similar trends were observed with the established RIs of UA and Cys C (Table 1), in which the lower and upper limits were much higher in the third trimester than those in the first trimester. Meanwhile, the indirect RI estimation method (the Hoffmann method), which was developed for relatively large data set and allowed mixing with small group of “unhealthy subjects”, was implemented with the first and third trimester RFTs data from the population pool(n = 16489). As shown in Table 1, there was no significant difference between the RIs of healthy pregnant women and those calculated by the Hoffman method (absolute difference % < RCV %).

**Table 1:** Reference interval of plasma renal tests during the first- and third-trimester.

	Direct observational method	Hoffmann method	Absolute difference, %	RCV, %
BUN-a (mmol/L)	1.87–4.59	1.87–4.61	0.00–0.43	34.7
BUN-b (mmol/L)	1.78–4.38	1.79–4.61	0.55–4.99	
UA-a (μmol/L)	126.57–295.10	132.50–317.83	4.48–7.15	24.0
UA-b (μmol/L)	162.22–375.40	164.40–400.59	1.33–6.29	
Cre-a (μmol/L)	34.40–57.90	34.91–58.57	1.46–1.14	13.4
Cre-b (μmol/L)	33.71–57.28	33.51–59.21	0.60–3.26	
Cys C-a (mg/ml)	0.43–0.76	0.44–0.77	2.27–1.30	13.8
Cys C-b (mg/ml)	0.59–1.29	0.60–1.35	1.67–4.44	

Abbreviations: BUN, Blood urea nitrogen; UA, Uric acid; Crea, Creatinine; Cys C, cystatin C; -a: first trimester; -b: third trimester.

### 3.2 Predictive values of RFTs for pregnant complications and adverse outcomes

To further investigate the associations of abnormal renal function and adverse pregnancy outcomes in the logistic regression with age and pre-pregnancy BMI as covariates, the ORs were calculated using the cut-offs set around the upper reference limits (URLs) for BUN, UA, Crea and Cys C (n = 16489). As shown in Table 2, the first- or third-trimester RFTs displayed diverse impacts on the risks of development of variable perinatal adverse outcomes. For instance, a strong positive association was observed between the third-trimester Cys C and the risk of PPH, with an OR of 4.11(95 %CI, 3.07–5.49) when Cys C was at or higher than 120 % of its URL. Similar positive associations were found in the UA regression analysis with the first-trimester GH and third-trimester PPH. Further, A rather stably increased and mild risk of GDM (ORs ranged from 1.59 to 1.81) and PE (ORs ranged from 1.58 to 2.01) was seen when the first-trimester UA was between 80 % and 120 % of its URL. Lastly, increased Crea was associated with a moderately increased risk of PPH development in the cohort.

**Table 2:** Logistic regression analysis of the risk of serum renal markers during pregnancy for pregnancy complications and adverse perinatal outcomes.

	BUN		UA		Crea		Cys C	
	OR (95 % CI)	p value	OR (95 % CI)	p value	OR (95 % CI)	p value	OR (95 % CI)	p value
ICP*								
80 % URL	1.13(0.44–2.90)	0.79	1.51(0.77–2.96)	0.24	0.90(0.49–1.66)	0.74	2.04(1.10–3.76)	0.02
85 % URL	0.71(0.17–2.96)	0.64	0.93(0.40–2.18)	0.87	1.06(0.55–2.01)	0.87	2.76(1.47–5.18)	<0.01
90 % URL	0.57(0.08–4.16)	0.58	0.78(0.27–2.29)	0.66	1.24(0.59–2.60)	0.57	2.59(1.23–5.49)	0.01
95 % URL	0	0.98	0.59(0.14–2.54)	0.48	1.06(0.38–2.99)	0.91	2.72(1.05–7.03)	0.04
100 % URL	0	0.99	0.44(0.06–3.33)	0.43	1.15(0.28–4.80)	0.85	2.16(0.51–9.08)	0.29
105 % URL	0	0.99	0.71(0.09–5.36)	0.74	0	0.99	0	0.99
110 % URL	0	0.99	1.12(0.15–8.55)	0.92	0	0.99	0	0.99
115 % URL	0	0.99	1.66(0.21–12.82)	0.63	0	0.99	0	0.99
120 % URL	0	0.99	2.45(0.32–19.02)	0.39	0	0.99	0	0.99
GDM*								
80 % URL	0.88(0.72–1.67)	0.19	1.70(1.50–1.94)	<0.01	0.87(0.77–0.98)	0.02	0.97(0.86–1.11)	0.70
85 % URL	0.83(0.65–1.07)	0.15	1.60(1.39–1.84)	<0.01	0.83(0.73–0.94)	<0.01	1.00(0.87–1.16)	1.00
90 % URL	0.95(0.71–1.29)	0.76	1.64(1.41–1.92)	<0.01	0.84(0.72–0.98)	0.03	1.01(0.84–1.22)	0.89
95 % URL	0.92(0.62–1.36)	0.66	1.59(1.33–1.90)	<0.01	0.83(0.68–1.02)	0.08	0.96(0.75–1.22)	0.72
100 % URL	0.86(0.51–1.45)	0.57	1.71(1.40–2.09)	<0.01	0.88(0.67–1.18)	0.40	0.93(0.65–1.31)	0.66
105 % URL	0.79(0.38–1.65)	0.54	1.70(1.34–2.15)	<0.01	0.95(0.63–1.43)	0.80	0.95(0.59–1.54)	0.83
110 % URL	0.56(0.17–1.81)	0.33	1.67(1.27–2.20)	<0.01	0.85(0.45–1.61)	0.61	1.14(0.61–2.12)	0.68
115 % URL	1.03(0.31–3.42)	0.96	1.74(1.27–2.39)	<0.01	0.89(0.34–2.25)	0.77	1.08(0.51–2.28)	0.84
120 % URL	2.09(0.59–7.32)	0.25	1.81(1.26–2.61)	<0.01	1.06(0.30–3.77)	0.93	1.41(0.64–3.13)	0.40
GH*								
80 % URL	1.54(1.11–2.13)	<0.01	1.43(1.12–1.83)	<0.01	0.88(0.71–1.11)	0.28	1.03(0.81–1.31)	0.83
85 % URL	1.55(1.04–2.30)	0.03	1.43(1.10–1.86)	<0.01	0.92(0.72–1.17)	0.49	1.09(0.83–1.42)	0.55
90 % URL	1.19(0.69–2.05)	0.53	1.49(1.12–1.99)	<0.01	1.00(0.75–1.32)	0.99	1.17(0.84–1.63)	0.35
95 % URL	0.86(0.38–1.94)	0.71	1.52(1.10–2.10)	0.01	1.18(0.84–1.67)	0.34	0.69(0.40–1.18)	0.17
100 % URL	0.48(0.12–1.94)	0.30	1.50(1.04–2.17)	0.03	1.14(0.69–1.87)	0.61	0.64(0.30–1.38)	0.25
105 % URL	0.94(0.23–3.83)	0.93	1.80(1.20–2.69)	<0.01	1.05(0.49–2.25)	0.91	0.17(0.02–1.23)	0.08
110 % URL	1.96(0.47–8.09)	0.35	1.76(1.10–2.80)	0.02	1.44(0.52–3.94)	0.48	0.33(0.04–2.38)	0.27
115 % URL	1.65(0.23–12.17)	0.62	2.02(1.22–3.36)	<0.01	0.73(0.10–5.36)	0.76	0.47(0.06–3.48)	0.46
120 % URL	0	0.98	2.23(1.26–3.94)	<0.01	1.67(0.22–12.67)	0.62	0.62(0.08–4.57)	0.64
PPH*								
80 % URL	1.07(0.89–1.29)	0.47	1.11(0.98–1.27)	0.11	0.94(0.84–1.06)	0.33	1.13(1.00–1.28)	0.05
85 % URL	1.01(0.80–1.28)	0.94	1.15(0.99–1.33)	0.07	0.98(0.87–1.11)	0.78	1.14(0.99–1.31)	0.07
90 % URL	1.03(0.77–1.38)	0.82	1.27(1.08–1.50)	<0.01	1.04(0.90–1.21)	0.58	1.25(1.05–1.49)	0.01
95 % URL	1.09(0.75–1.58)	0.64	1.19(0.98–1.44)	0.08	1.06(0.87–1.28)	0.58	1.39(1.11–1.75)	<0.01
100 % URL	1.24(0.78–1.95)	0.36	1.21(1.02–1.46)	0.03	1.08(0.82–1.41)	0.60	1.27(0.91–1.76)	0.16
105 % URL	1.31(0.71–2.38)	0.39	1.48(1.15–1.91)	<0.01	1.71(1.21–2.42)	<0.01	1.29(0.82–2.03)	0.28
110 % URL	1.49(0.67–3.29)	0.33	1.29(0.95–1.77)	0.11	2.22(1.37–3.59)	<0.01	1.20(0.64–2.27)	0.57
115 % URL	1.03(0.32–3.38)	0.96	1.37(0.95–1.96)	0.09	2.81(1.43–5.56)	<0.01	0.58(0.21–1.61)	0.30
120 % URL	1.29(0.30–5.60)	0.73	1.36(0.89–2.07)	0.16	2.11(0.70–6.36)	0.19	0.80(0.29–2.24)	0.67
PE*								
80 % URL	1.00(0.76–1.30)	0.97	1.60(1.34–1.91)	<0.01	1.06(0.90–1.25)	0.47	1.14(0.96–1.35)	0.14
85 % URL	0.90(0.63–1.28)	0.56	1.67(1.39–2.01)	<0.01	1.10(0.93–1.31)	0.25	1.17(0.97–1.41)	0.10
90 % URL	0.85(0.54–1.33)	0.48	1.72(1.41–2.10)	<0.01	1.03(0.84–1.26)	0.78	1.17(0.92–1.47)	0.20
95 % URL	1.14(0.68–1.92)	0.61	1.68(1.34–2.09)	<0.01	1.03(0.80–1.34)	0.81	1.00(0.73–1.38)	0.99
100 % URL	1.78(1.02–3.11)	0.04	1.58(1.23–2.03)	<0.01	1.03(0.72–1.49)	0.85	0.86(0.54–1.36)	0.51
105 % URL	1.72(0.79–3.76)	0.17	1.81(1.36–2.40)	<0.01	1.06(0.63–1.79)	0.83	1.00(0.54–1.80)	0.97
110 % URL	1.50(0.46–4.88)	0.50	1.89(1.37–2.60)	<0.01	1.29(0.62–2.70)	0.50	0.67(0.26–1.72)	0.40
115 % URL	0.85(0.11–6.26)	0.87	2.01(1.40–2.88)	<0.01	1.70(0.65–4.42)	0.28	1.00(0.38–2.63)	1.00
120 % URL	1.50(0.20–11.53)	0.70	1.88(1.23–2.86)	<0.01	2.23(0.62–7.93)	0.22	1.06(0.36–3.14)	0.92
Macrosomia*								
80 % URL	0.80(0.53–1.21)	0.30	0.81(0.62–1.06)	0.13	0.88(0.69–1.11)	0.27	0.77(0.59–1.00)	0.05
85 % URL	0.91(0.55–1.50)	0.72	0.84(0.63–1.14)	0.26	1.02(0.80–1.30)	0.86	0.70(0.51–0.96)	0.03
90 % URL	0.67(0.33–1.36)	0.27	1.08(0.79–1.49)	0.62	0.85(0.62–1.15)	0.28	0.61(0.40–0.94)	0.03
95 % URL	0.57(0.21–1.55)	0.27	0.99(0.68–1.43)	0.94	0.70(0.46–1.09)	0.11	0.52(0.28–0.97)	0.04
100 % URL	0.49(0.12–1.99)	0.32	0.74(0.46–1.18)	0.20	1.04(0.61–1.76)	0.89	0.58(0.25–1.32)	0.19
105 % URL	0.95(0.23–3.86)	0.94	1.05(0.64–1.72)	0.85	1.25(0.61–2.56)	0.55	0.56(0.18–1.79)	0.33
110 % URL	1.93(0.47–7.99)	0.36	0.80(0.43–1.51)	0.50	1.09(0.34–3.46)	0.89	0.35(0.05–2.55)	0.30
115 % URL	1.64(0.22–12.11)	0.63	0.93(0.47–1.87)	0.85	0.77(0.10–5.63)	0.80	0.50(0.07–3.67)	0.50
120 % URL	3.02(0.40–22.85)	0.29	0.85(0.37–1.97)	0.70	0	0.97	0.68(0.09–4.99)	0.70
PPH**								
80 % URL	1.07(0.93–1.24)	0.34	1.38(1.20–1.59)	<0.01	0.93(0.83–1.04)	0.21	1.70(1.50–1.92)	<0.01
85 % URL	1.23(1.04–1.45)	0.01	1.57(1.34–1.84)	<0.01	0.99(0.87–1.12)	0.87	1.96(1.72–2.24)	<0.01
90 % URL	1.20(0.98–1.46)	0.08	1.96(1.65–2.34)	<0.01	1.08(0.93–1.26)	0.29	2.11(1.82–2.43)	<0.01
95 % URL	1.21(0.95–1.54)	0.12	2.06(1.68–2.52)	<0.01	1.23(1.03–1.48)	0.03	2.32(1.97–2.73)	<0.01
100 % URL	1.36(1.02–1.81)	0.04	2.29(1.80–1.69)	<0.01	1.45(1.15–1.84)	<0.01	2.51(2.09–3.01)	<0.01
105 % URL	1.45(1.03–2.03)	0.03	2.35(1.77–3.11)	<0.01	1.70(1.26–2.30)	<0.01	2.89(2.37–3.54)	<0.01
110 % URL	1.49(0.98–2.28)	0.06	2.15(1.53–3.03)	<0.01	1.56(1.02–2.39)	0.04	3.10(2.46–3.91)	<0.01
115 % URL	1.55(0.94–2.56)	0.08	2.63(1.77–3.91)	<0.01	2.01(1.20–3.35)	<0.01	3.56(2.74–4.61)	<0.01
120 % URL	1.75(0.97–3.16)	0.06	2.75(1.74–4.35)	<0.01	1.94(0.98–3.85)	0.06	4.11(3.07–5.49)	<0.01
Macrosomia**								
80 % URL	0.50(0.35–0.73)	<0.01	0.52(0.36–0.74)	<0.01	0.63(0.50–0.81)	<0.01	0.80(0.61–1.06)	0.12
85 % URL	0.46(0.29–0.75)	<0.01	0.53(0.35–0.82)	<0.01	0.60(0.45–0.80)	<0.01	0.98(0.72–1.33)	0.89
90 % URL	0.43(0.24–0.79)	<0.01	0.45(0.26–0.79)	<0.01	0.57(0.40–0.82)	<0.01	1.02(0.72–1.45)	0.90
95 % URL	0.37(0.16–0.83)	0.02	0.58(0.31–1.06)	0.08	0.54(0.33–0.88)	0.01	0.91(0.59–1.39)	0.65
100 % URL	0.19(0.05–0.76)	0.02	0.81(0.43–1.55)	0.53	0.43(0.20–0.91)	0.03	0.81(0.48–1.37)	0.43
105 % URL	0.29(0.07–1.18)	0.08	0.59(0.24–1.44)	0.24	0.58(0.24–1.43)	0.24	0.98(0.56–1.73)	0.95
110 % URL	0	0.97	0.50(0.16–1.57)	0.24	0.70(0.22–2.20)	0.54	0.74(0.35–1.58)	0.44
115 % URL	0	0.97	0.76(0.24–2.42)	0.65	0.38(0.05–2.75)	0.34	0.58(0.22–1.58)	0.29
120 % URL	0	0.97	0.70(0.17–2.86)	0.62	0.68(0.09–4.93)	0.70	0.59(0.19–1.84)	0.36

*: first trimester; **: third trimester; OR: odds ratio; CI: confidence interval; URL: upper reference interval limit; ICP: intrahepatic cholestasis of pregnancy; GDM: gestational diabetes mellitus; GH: gestational hypertension; PE: preeclampsia; PPH: postpartum hemorrhage; BUN, blood urea nitrogen; UA, uric acid; Crea, creatinine; Cys C, cystatin C.

Demographic data for age, pre-pregnancy BMI, and basic statistics for RFT are summarized in Table 3. Specifically, the third-trimester BUN and Crea levels were decreased in the Macrosomia group than in the control group (*p* < 0.001); the first-trimester UA levels were significantly increased in the GH, GDM, and PE groups than in the control group (*p* < 0.001). Both early and late gestational UA levels were significantly higher in the PPH group than in the control group (*p* < 0.001). Interestingly, marginally increased levels of the first-trimester Cys C were observed in the patients developing GDM, PE, and PPH, and the third-trimester Cys C was also mildly increased in the PPH group (*p* < 0.001).

**Table 3:** Demographic data for the pregnant women included.

	Age	BMI (Pre-pregnancy)	BUN (mmol/L)	UA (μmol/L)	Crea (μmol/L)	Cys C(mg/ml)
GH positive^a^ (N = 317)	31(29–35)	23.23(20.76–26.17)	2.88(2.46–3.40)	224.7(192.00–261.6)	44.9(41.30–49.60)	0.59(0.53–0.64)
GH negative^a^ (N = 16172)	31(29–34)	21.23(19.53–23.44)	2.93(2.53–3.41)	204.4(177.20–236.70)	45.3(41.40–49.30)	0.58(0.53–0.64)
*P* value	0.176	<0.001	0.544	<0.001	0.999	0.148
GDM positive^a^ (N = 1239)	33(30–36)	23.03(20.90–25.63)	2.95(2.55–3.41)	226.8(194.40–261.40)	45.20(41.60–49.20)	0.59(0.54–0.65)
GDM negative^a^ (N = 15250)	31(29–34)	21.12(19.49–23.28)	2.93(2.52–3.4)	203.3(176.50–235.00)	45.30(41.40–49.30)	0.58(0.53–0.64)
*P* value	<0.001	<0.001	0.193	<0.001	0.870	<0.001
PE positive^a^ (N = 631)	32(29–35)	24.03(21.23–26.95)	2.97(2.52–3.42)	231.7(199.05–270.50)	46.00(41.40–49.30)	0.60(0.54–0.66)
PE negative^a^ (N = 15858)	31(29–35)	21.22(19.53–23.34)	2.93(2.52–3.4)	203.8(176.80–235.80)	45.20(41.40–50.20)	0.58(0.53–0.64)
*P* value	<0.001	<0.001	0.824	<0.001	0.040	<0.001
ICP positive^a^ (N = 42)	31.5(29–35)	20.89(19.53–23.73)	2.98(2.48–3.51)	207.75(179.80–238.50)	44.50(38.80–50.20)	0.59(0.51–0.68)
ICP negative^a^ (N = 16447)	31(29–34)	21.26(19.53–23.44)	2.93(2.52–3.40)	204.75(177.50–237.10)	45.3(41.40–49.30)	0.58(0.53–0.64)
*P* value	0.476	0.580	0.784	0.404	0.555	0.564
Macrosomia positive^a^ (N = 294)	32(30–35)	22.93(21.09–25.40)	2.93(2.46–3.40)	212.10(182.30–243.10)	45.15(41.20–49.30)	0.58(0.53–0.64)
Macrosomia negative^a^ (N = 16195)	31(29–34)	21.23(19.53–23.44)	2.93(2.53–3.41)	204.7(177.40–237.00)	45.30(41.40–49.30)	0.58(0.53–0.64)
*P* value	<0.001	<0.001	0.295	0.026	0.631	0.960
Macrosomia positive^b^ (N = 294)	32(30–35)	22.93(21.09–25.40)	2.68(2.30–3.08)	249.70(215.80–282.70)	42.75(39.40–47.30)	0.88(0.77–1.01)
Macrosomia negative^b^ (N = 16195)	31(29–34)	21.23(19.53–23.44)	2.88(2.46–3.37)	256.20(222.60–295.05)	44.50(40.60–48.90)	0.89(0.78–1.03)
*P* value	<0.001	<0.001	<0.001	0.017	<0.001	0.276
PPH positive^a^ (N = 1251)	32 (29–35)	22.04 (20.06–24.50)	2.95(2.56–3.40)	211.7(181.85–245.85)	45.30(41.50–49.40)	0.59(0.54–0.65)
PPH negative^a^ (N = 15238)	31 (29–34)	21.23 (19.53–23.38)	2.93(2.52–3.41)	204.2(177.10–236.40)	45.30(41.40–49.30)	0.58(0.53–0.64)
*P* value	<0.001	<0.001	0.603	<0.001	0.561	<0.001
PPH positive^b^ (N = 1251)	32 (29–35)	22.04 (20.06–24.50)	2.89(2.46–3.38)	265.90(228.65–308.80)	44.40(40.10–49.20)	0.94(0.81–1.12)
PPH negative^b^ (N = 15238)	31 (29–34)	21.23 (19.53–23.38)	2.87(2.45–3.36)	255.40(221.90–293.70)	44.50(40.60–48.90)	0.89(0.78–1.02)
*P* value	<0.001	<0.001	0.442	<0.001	0.393	<0.001

All values were presented as median (25th-75th percentile). a: first trimester; b: third trimester; GH: gestational hypertension; GDM: gestational diabetes mellitus; PE: preeclampsia; ICP: intrahepatic cholestasis of pregnancy; PPH: postpartum hemorrhage; BMI: body mass index; BUN: blood urea nitrogen; UA: uric acid; Cre: creatinine; Cys C: cystain C.

## 4 Discussion

As sensitive and widely used renal markers during pregnancy, the serum concentrations of BUN, UA, Crea, and Cys C are varied along with the gestational age due to physiological changes in renal function [1,18]. By monitoring renal function markers in the first- and third-trimester healthy pregnancy, the levels of BUN and Crea were decreased and the levels of UA and Cys C were increased in the third-trimester (Table 1 and Fig. 1). These findings were consistent with what has been previously reported [1,4,19]. Both blood volume and eGFR were found to stay at a high level throughout pregnancy, causing both serum Crea and BUN levels to decrease [1,20]. Meanwhile, increased serum Cys C and UA concentrations in late pregnancy may be associated with glomerular swelling caused by high blood volume and an altered renal handling level of urate [4,21]. Together, our study has proven the necessity and importance of establishing trimester-specific RIs of RFTs for pregnancies.

In our logistic regression analysis, the high levels of UA, Crea, and Cys C in late pregnancy were associated with an increased risk of PPH. Similarly, there were several studies that showed a positive relationship between abnormal renal function and higher bleeding tendencies [8,9,22,23]. For example, Yuan et al. reported a significantly increased risk of symptomatic intracranial hemorrhage after endovascular treatment in patients with elevated concentrations of serum UA after adjusting for a series of potential confounders [18]. For pregnant women, a higher UA level leads to uterine vascular endothelial damage and increases atherosclerotic lesions on the vessel walls by releasing massive inflammatory mediators, therefore, higher bleeding tendencies [23]. Additionally, a case-control study by Li Ni et al. [9] indicated that Cys C was a strong predictor of cardiovascular events and mortality risk. Excess serum Cys C may disturb the balance of proteolytic and anti-proteolytic activity, which directly affects the remodeling process of the vascular wall and leads to the development of PPH [22].

Interestingly, increased UA in early pregnancy was found to be associated with the moderately increased risks of GDM, GH, and PE, which is similar to those reported by other research groups [10,24]. For instance, Wolak et al. [10] demonstrated that increased UA level is an independent risk factor for both GDM and mild PE. In Areda et al.‘s study [24] pregnancy‐induced hypertension women were found to present hyperuricemia compared to normotensive pregnant women.

Further studies [[25], [26], [27]] showed that there was a causal effect between increased UA and insulin resistance in pregnancy. Nakagawa et al. [25] proposed that accumulated serum UA caused endothelial dysfunction and decreased secretion of nitric oxide (NO) by the endothelial cells. The effect of insulin on glucose uptake into cells in the skeletal muscle and adipose tissue relies on NO [26]. Thus, decreased NO leads to decreased glucose uptake and the development of insulin resistance, resulting in GDM. Moreover, insulin resistance might be bound up with increased blood pressure due to increased adrenergic tone, which may inhibit glucose metabolism and increase vascular resistance, potentially leading to GH [19]. On the other hand, hyperuricemia is a key biochemical feature in PE [28,29]. This persistent hyperuricemia may reduce the NO produced by endothelial cells, which in turn hinders the invasion of the trophoblast, failing vascular remodeling of the placental bed resulting in PE [29].

Moderately increased risk of ICP development was seen in the pregnant women whose first-trimester Cys C levels were between 80 % and 95 % URL (Table 2). According to the study by Smolarczyk et al. [30], disturbances of biochemical functions of kidney glomeruli and tubules were revealed in women suffering from intrahepatic cholestasis in pregnancy. Meanwhile, Cys C, a potent inhibitor of cysteine proteinases, may participate in the development of fibrosis by inactivating cathepsins, which may be related to the progression of hepatic diseases [31]. Further, serum Cys C concentrations in the intrahepatic cholestasis patients were found significantly elevated than in the controls [32]. Thus, Cys C may act as a sensitive marker in monitoring liver function during pregnancy.

Our study was based on a relatively large population-based cohort (n = 16489), rendering ample statistical power for the subsequent regression analysis. However, as this was a single-center study with narrow representation of pregnant women, the application of the derived RIs of RFTs was limited. In addition, for routine antenatal check-up in our institute, the RFTs were not regularly performed in pregnant women at their second trimester, leading to the missing of the RIs in this gestational period in our study. A future study involving a multicentric population is warranted to take wider geographical, ethnic, and socioeconomic backgrounds into consideration.

## 5 Conclusion

In summary, with the established trimester-specific RIs of RFTs, diverse associations between adverse perinatal outcomes and abnormal renal function were revealed.

## Funding

This work was supported by Beijing Health Technologies Promotion Program (BHTPP2022017) and Beijing Hospitals Authority Clinical Medicine Development of Special Funding Support (code: ZYLX202120). The funding body did not take part in the design of the study, the collection, analysis and interpretation of the data, or manuscript writing.

## Author statement

The manuscript has been read and approved by all the authors. It has not been published, submitted, or accepted for publication elsewhere. And there is no financial interest or conflicts of interest to disclose.

## Declaration of competing interest

This work was supported by Beijing Health Technologies Promotion Program (BHTPP2022017). The funding body did not take part in the design of the study, the collection, analysis and interpretation of the data, or manuscript writing. There is no financial interest or conflicts of interest to disclose.

## Data Availability

Data will be made available on request.
