# Supplementary material for: Establishment of trimester-specific reference intervals of renal function tests and their predictive values in pregnant complications and perinatal outcomes: A population-based cohort study
Source: Pract Lab Med. 2023 Oct 16;37:e00342. doi: 10.1016/j.plabm.2023.e00342 (PMC10590743; doi:10.1016/j.plabm.2023.e00342)
Supplement: Multimedia component 1 [file mmc1.docx]

**Supplemental Table 1** Diagnosis criteria of pregnancy complications and adverse prenatal outcomes

|  |  |  |
| --- | --- | --- |
| Diseases | Definition |  |
| GH | Defined as blood pressure elevation [systolic blood pressure ≥ 140 mmHg or diastolic blood pressure ≥ 90 mmHg] at > 20 weeks’ gestation in the absence of proteinuria (1). |  |
| GDM | Before an oral glucose tolerance test (OGTT), every participant was requested for a 50 g glucose challenge test and serum glucose levels were assayed 1 h later. Subjects with positive results (glucose levels ≥7.8 mmol/L) were required to undergo a 75 g OGTT. Serum glucose levels during OGTT were measured at 0, 1 and 2 h, respectively. The normal values were fasting glucose <5.1 mmol/L, 1-h glucose <10.0 mmol/L and 2-h glucose <8.5 mmol/L. If one or more values equaled or exceeded the above thresholds, women were diagnosed as having GDM (2). |  |
| PE | New-onset hypertension (systolic blood pressure ≥140 mmHg or diastolic blood pressure ≥90 mmHg) and new-onset proteinuria (300 mg of protein in 24 h or a urine protein/creatinine ratio of 0.3 mg/dl) after 20 weeks of gestation, in a previously normotensive woman (1). |  |
| ICP | ICP is a pregnancy-specific disorder typically occurs in the third trimester characterized by pruritus and jaundice. Confirmation of diagnosis relied on abnormal liver function tests and raised maternal serum bile acids. Abnormal liver function tests included elevated levels of alanine aminotransferase, aspartate aminotransferase and/or gamma-glutamyl transpeptidase. The upper limits of total serum bile acids were 10–14 μmoles/L in postprandial state and 6–10 micromoles/L in fasting state. Exceeding the upper limits was an important diagnostic basis of ICP (3) |  |
| Macrosomia | Fetal birth weight ≥ 4000 g, regardless of gestational age (4). |  |
| PPH | Cumulative blood loss of ≥1,000 mL OR blood loss accompanied by signs and symptoms of hypovolemia within 24 hours following the birth process (5). |  |
| GH: gestational hypertension; GDM: gestational diabetes mellitus; PE: preeclampsia; ICP: intrahepatic cholestasis of pregnancy; PTB: preterm birth; FGR: fetal growth restriction; PPH: postpartum hemorrhage. | |  |

**References**

1. Hypertension in pregnancy. Report of the American College of Obstetricians and Gynecologists' task force on hypertension in pregnancy. Obstet Gynecol. 2013;122(5):1122–31.

2. World Health Organization. Diagnostic criteria and classification of hyperglycemia first detected in pregnancy: a World Health Organization Guideline. Diabetes Res Clin Pract. 2014;103(3):341–63.

3. Williamson C, Geenes V. Intrahepatic cholestasis of pregnancy. Obstet Gynecol. 2014;124(1):120–33.

4. Macrosomia: ACOG Practice Bulletin, Number 216. Obstet Gynecol. 2020;135(1):e18-e35.

5 Practice Bulletin No. 183: Postpartum Hemorrhage. Committee on Practice Bulletins-Obstetrics. Obstet Gynecol. 2017;130(4):e168-e186
